# Reveals of New Candidate Active Components in Hemerocallis Radix and Its Anti-Depression Action of Mechanism Based on Network Pharmacology Approach

**DOI:** 10.3390/ijms21051868

**Published:** 2020-03-09

**Authors:** Hsin-Yi Lin, Jen-Chieh Tsai, Lung-Yuan Wu, Wen-Huang Peng

**Affiliations:** 1 School of Chinese Pharmaceutical Sciences and Chinese Medicine Resources, China Medical University, No. 91, Hsueh-Shih Road, Taichung 40402, Taiwan; 2 Department of Medicinal Botanicals and Health Applications Da-Yeh University, No.168, University Rd., Dacun, Changhua 51591, Taiwan; jenchieh@mail.dyu.edu.tw; 3 Graduate Institute of Chinese Pharmaceutical Sciences, College of Pharmacy, China Medical University, No. 91, Hsueh-Shih Road, Taichung 40402, Taiwan; dr.wuly@gmail.com

**Keywords:** network pharmacology, *Hemerocallis* radix, depressive disorder, MAOA, MAOB, ESR1, vanillic acid, anthraquinone, kaempferol

## Abstract

The global depression population is showing a significant increase. *Hemerocallis fulva* L. is a common Traditional Chinese Medicine (TCM). Its flower buds are known to have ability to clear away heat and dampness, detoxify, and relieve depression. Ancient TCM literature shows that its roots have a beneficial effect in calming the spirit and even the temper in order to reduce the feeling of melancholy. Therefore, it is inferred that the root of *Hemerocallis fulva* L. can be used as a therapeutic medicine for depression. This study aims to uncover the pharmacological mechanism of the antidepressant effect of *Hemerocallis* Radix (HR) through network pharmacology method. During the analysis, 11 active components were obtained and screened using ADME—absorption, distribution, metabolism, and excretion— method. Furthermore, 267 HR targets and 740 depressive disorder (DD) targets were gathered from various databases. Then protein–protein interaction (PPI) network of HR and DD targets were constructed and cluster analysis was applied to further explore the connection between the targets. In addition, gene ontology (GO) enrichment and pathway analysis was applied to further verify that the biological process related to the target protein is associated with the occurrence of depression disorder. In conclusion, the most important bioactive components—anthraquinone, kaempferol, and vanillic acid—can alleviate depression symptoms by regulating MAOA, MAOB, and ESR1. The proposed network pharmacology strategy provides an integrating method to explore the therapeutic mechanism of multi-component drugs on a systematic level.

## 1. Introduction

The burden of depression and other mental health conditions is on the rise globally. Depression alone accounts for 4.3% of the global burden of disease with more than 300 million people affected. People with mental health disorders often have higher rates of mortality, 40% to 60% greater than the general population. Therefore, the World Health Organization (WHO) ranked depression as the second most important disease to be considered in 2020 [1].

Mental disorders often affect, and are affected by, other diseases. If worse comes to worst, suicide is the case. Depression and suicide are significant public health concerns, with over 40,000 Americans dying by suicide each year [2]. Beyond the lives lost to suicide, death by suicide has significant emotional and economic costs, resulting in approximately $44.6 billion a year in combined medical and work loss costs in the United States alone [2]. As such, suicide—and factors that may increase the risk for suicide, including depression—is a serious public health concern that warrants extensive empirical investigation. The number of depression patients also grows slowly every year in Taiwan. According to the Ministry of Health and Welfare (MOHW), there were 1.3 million people—accounted for 6% of the Taiwanese population—using antidepressive drugs. And more than 60% were above 45 years old [3]. 

Guidelines on the duration of antidepressant prescriptions differ in detail, but in general, about 6 to 12 months as WHO and NICE (National Institute for Health and Care Excellence) recommended. However, patients with long-term antidepressant treatment have trade-offs in adherence that might lead to discontinuation of these drugs. Taiwanese, especially elders, has a special place in their heart for Traditional Chinese Medicine (TCM). Many of us believe that TCM has benefits toward human health. In addition, more than 6.4 million people went to a Chinese medication clinic, 3% of them have been diagnosed with mental or behavioral disorders [4]. As a critical component of complementary and alternative medicine, TCM plays an important role in treating depression.

*Hemerocallis fulva* L.—Orange daylily or Nepenthe—is a common TCM. A species of perennial flowering daylily in genus *Hemerocallis* (*Hemerocallidoideae* family). It is native to Himalaya, East Europe, China, Japan, and Korea [5,6,7,8], and was imported into Taiwan in 1661. It was first entered in the publication Supplement to Compendium of Materia Medica (Bencao Gangmu Shiyi). The root, seeding, and flower of *Hemerocallis fulva* are considered to have sweet, cool, and non-toxic properties and to be associated with the spleen, liver, and bladder meridians [9]. Its flower buds have heat-clearing, damp-draining, blood-cooling, and detoxifying properties, coursing the liver to relieve depression. It has been used in many therapeutic prescriptions since ancient times. Ancient TCM literature also showed that its roots have a beneficial effect in calming the spirit and even the temper, in order to reduce the feeling of melancholy [10,11]. Therefore, it is inferred that *Hemerocallis* Radix (HR)—the root of *Hemerocallis fulva* L.—can be used as a therapeutic medicine for depression or as an auxiliary medication.

**Figure 1:**
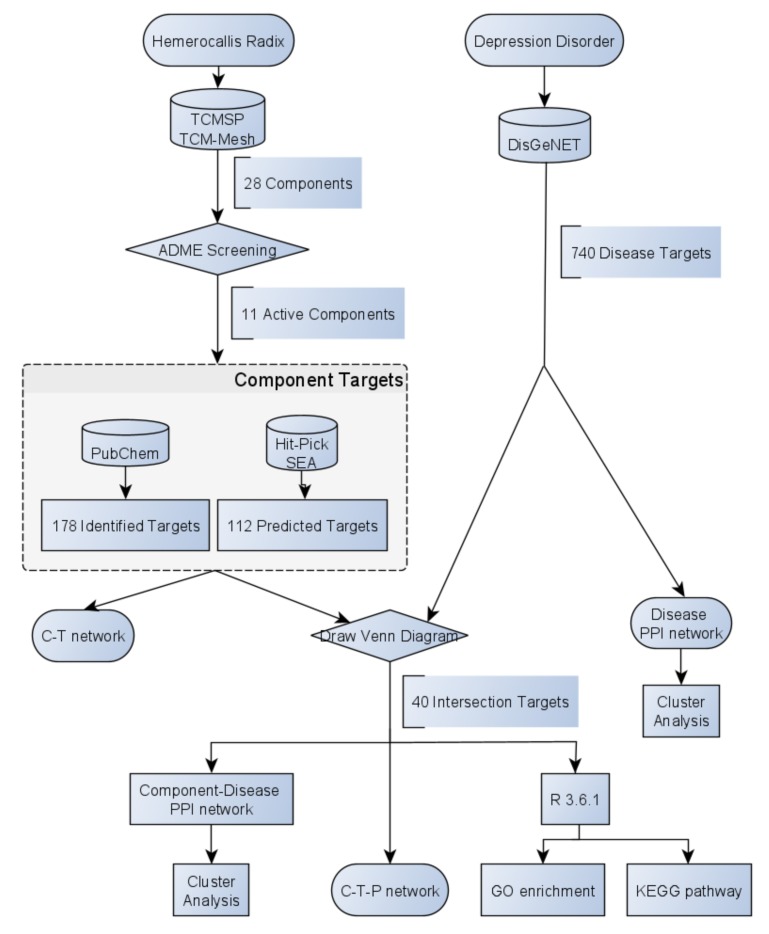
Flowchart of this study.

So far, several analytical methods, such as HPLC and LC-MS/MS has been reported to evaluate the effective compounds [12]. Nevertheless, there is no literature expounds on the underlying therapeutic mechanism of HR on DD so far. Consider the flaws of traditional experimental and analytical methods, it is difficult to uncover its association between herb-component-target-disease due to one of the greatest features of TCM system: multi-component and multi-target. Network pharmacology is an effective tool to expound the synergistic and potential mechanisms of the networks between component-target-disease and protein–protein interaction (PPI), it provides a new perspective on the therapeutic mechanisms of TCM. Therefore, the main purpose of this research is to identify effective target proteins of bioactive components in HR toward DD through network pharmacology, which is first introduced by Hopkins [13,14]. The work scheme of this research is shown in Figure 1, integrating pharmacokinetics synthesis screening, target identification, and network analysis.

## 2. Results

Based on the network pharmacology model we constructed, the therapeutic mechanisms of HR toward DD were clarified. HR components were collected from databases. Next, numerous ADME—absorption, distribution, metabolism, and excretion—methods were used to screen for potential active components. Then the obtained data were used for gene ontology (GO) and Kyoto Encyclopedia of Genes and Genomes (KEGG) analysis. All PPI networks will be further studied through cluster analysis. Finally, Component-Target-Pathway network is calculated and analyzed by the network visualization tool Cystoscape. 

### 2.1. Components in Hemerocallis Radix and Pharmacokinetic evaluation

Although every TCM herb contains multiple components, only a couple of them lives up to the standard of pharmacokinetic property. In this study, four ADME-related models were used to screen out the active components in HR, including oral bioavailability (OB), Caco-2 permeability (Caco-2), drug-likeness (DL), and GI absorption. Beyond that, all screen-out components should follow Lipinski’s rule of five. After this process, a few components that did not meet the ADME criteria were added back into the database because of their high bioactive or curative effect proved in previous studies. Therefore, a total of 28 components were collected through databases. All identified components were subjected to ADME screening, and 11 of the 28 passed the ADME criteria. That is to say, these are the active component of HR. The detail information is shown in Table 1.

### 2.2. Component-Target Network Construction

According to the pharmacokinetic evaluation of HR, aloe-emodin, α-boswellic acid, anthraquinone, β-boswellic acid, chrysophanol, colchicine, hemerocallone, kaempferol, puerarin, rhein, and vanillic acid were selected to be the active components of this herb, which were chosen in the following network pharmacology investigation. Thus, a network pharmacology approach was established to uncover the treatment mechanism of depression.

Three databases were engaged to assemble component-related targets. Total of 357 identified targets were collected through PubChem. 178 remain after deleting those overlapping targets. Among these components, chrysophanol, kaempferol, and aloe-emodin have the highest number of targets, where α-Boswellic acid and Hemerocallone have no identified target at all. For predicted targets, two databases were put into use. Total of 22 targets were predicted by HitPick under the condition of precision ≥ 50%. Twenty remains after deleting those overlapping the targets with colchicine having the highest number of targets, and hemerocallone has 0 predicted target. A total of 138 targets were predicted by SEA under the condition of MaxTc ≥ 0.5 which stands for the maximum tanimoto similarity between compounds. Total of 92 remain after deleting those overlapping the targets. Among these components, vanillic acid, kaempferol, and anthraquinone have the highest number of targets, whereas β-Boswellic acid, α-Boswellic acid, and Hemerocallone have less number of predicted targets.

**Figure 2:**
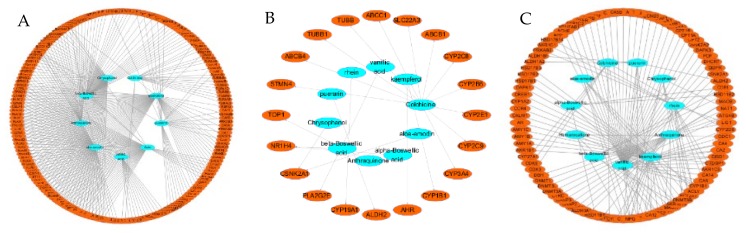

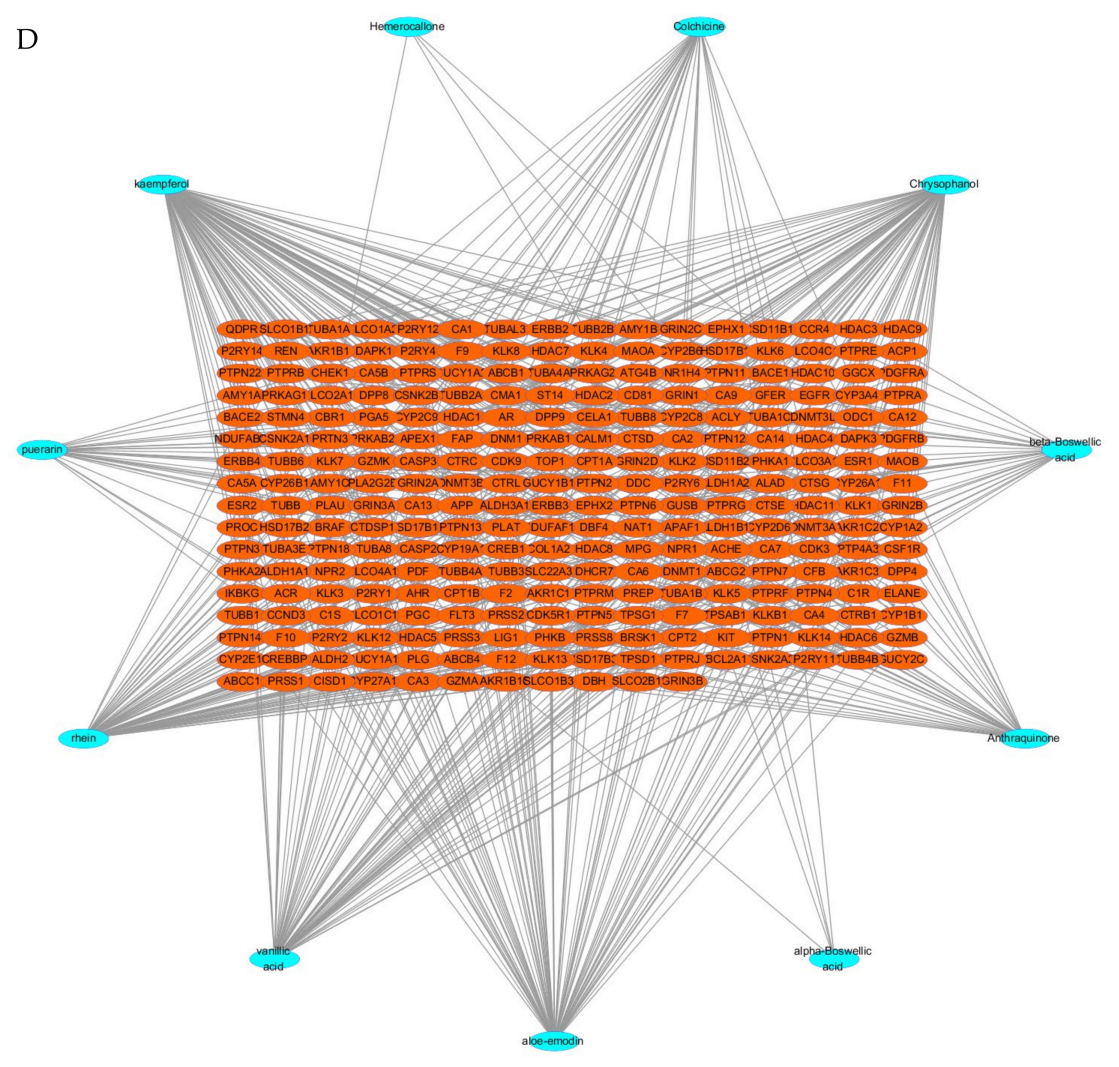
Component-target network of HR. The blue ellipse are active components of *Hemerocallis* Radix, and the orange eclipse nodes are the related targets. (**A**) The network of the identified targets. (**B**) The network of the predicted targets by HitPick. (**C**) The network of the predicted targets by similarity ensemble approach (SEA). (**D**) The network of all HR active component-related targets.

To explore the therapeutic mechanism of HR in the treatment of DD, 267 targets and 11 components were used to construct the component-target (C-T) network (Figure 2). All of these active components are related to multiple targets, resulting in 509 component-target associations between 11 active components and 267 targets. The average number of targets per component is 24.3, and the mean degree of components per target is 1.9. This clearly shows that HR fits the multi-component and multi-target characteristics of TCM. Aloe-emodin (HR01, degree = 99) has the highest number of targets, followed by chrysophanol (HR05, degree = 77), rhein (HR10, degree = 72), and kaempferol (HR08, degree = 71), indicating these components of HR have great possibility of becoming key components of treating depressive disorder.

### 2.3. Disease PPI Network Construction

Based on the result from gene database DisGeNET, there were a total of 740 candidate targets relevant to depressive disorder. Gene IDs of these targets were input into String, species were limited to “Homo sapiens,” and confidence score was set at 0.7. Then, it was imported into Cytoscape3.7.1 (http://www.cytoscape.org/) [15] to construct the network. After PPI was acquired, superfluous entries were removed and 709 nodes were found in the PPI network, which means there are 709 targets related to this specific disease.

**Figure 3:**
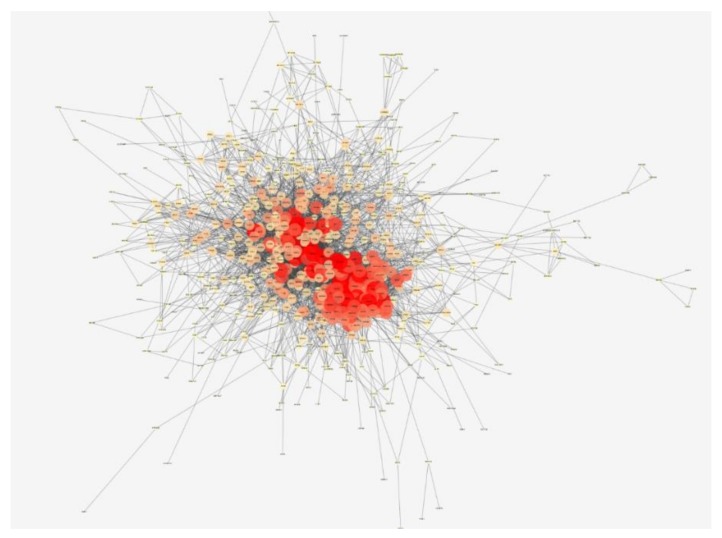
Protein–protein interaction (PPI) network of depressive disorder (DD). The closer, redder and the larger the nodes are, the higher the degree of freedom they have.

There are 709 nodes and 5506 edges in the DD target PPI network (Figure 3). The closer the nodes are, the more they are prone to show the color red and the larger the nodes are, the higher the degree of freedom they have. This demonstrates that these genes are closely related to other genes in this network, suggesting that these genes may play an important role in depressive disorder. Pathogenic factors may directly influence DD-related genes or indirectly influence DD-related genes by affecting these genes, thereby affecting the development of DD, which suggests that these genes may be the key to the treatment development of depressive disorder. The top 10 proteins with the highest degree of freedom are APP, GNB1, GNB3, PIK3CA, AKT1, AGT, IL6, TP53, EGFR, and INS. The respective degrees of freedom are 119, 113, 103, 100, 97, 87, 87, 86, 84, and 84.

### 2.4. Clusters of DD Target Network

**Figure 4:**
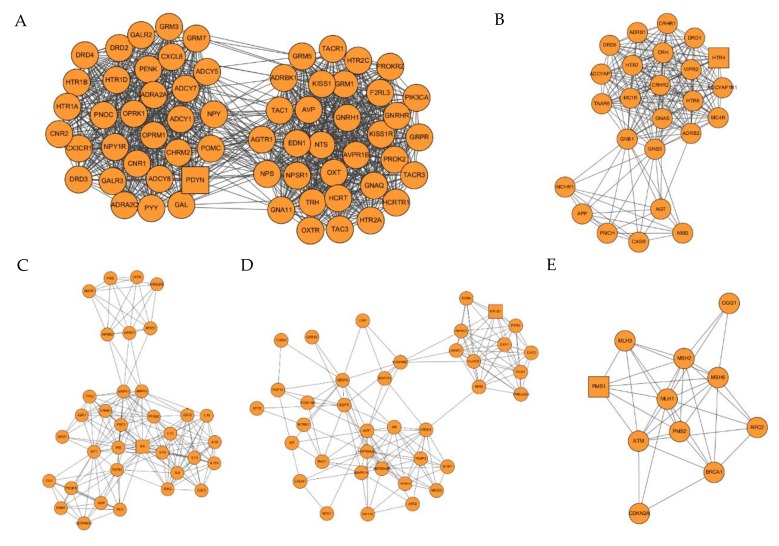

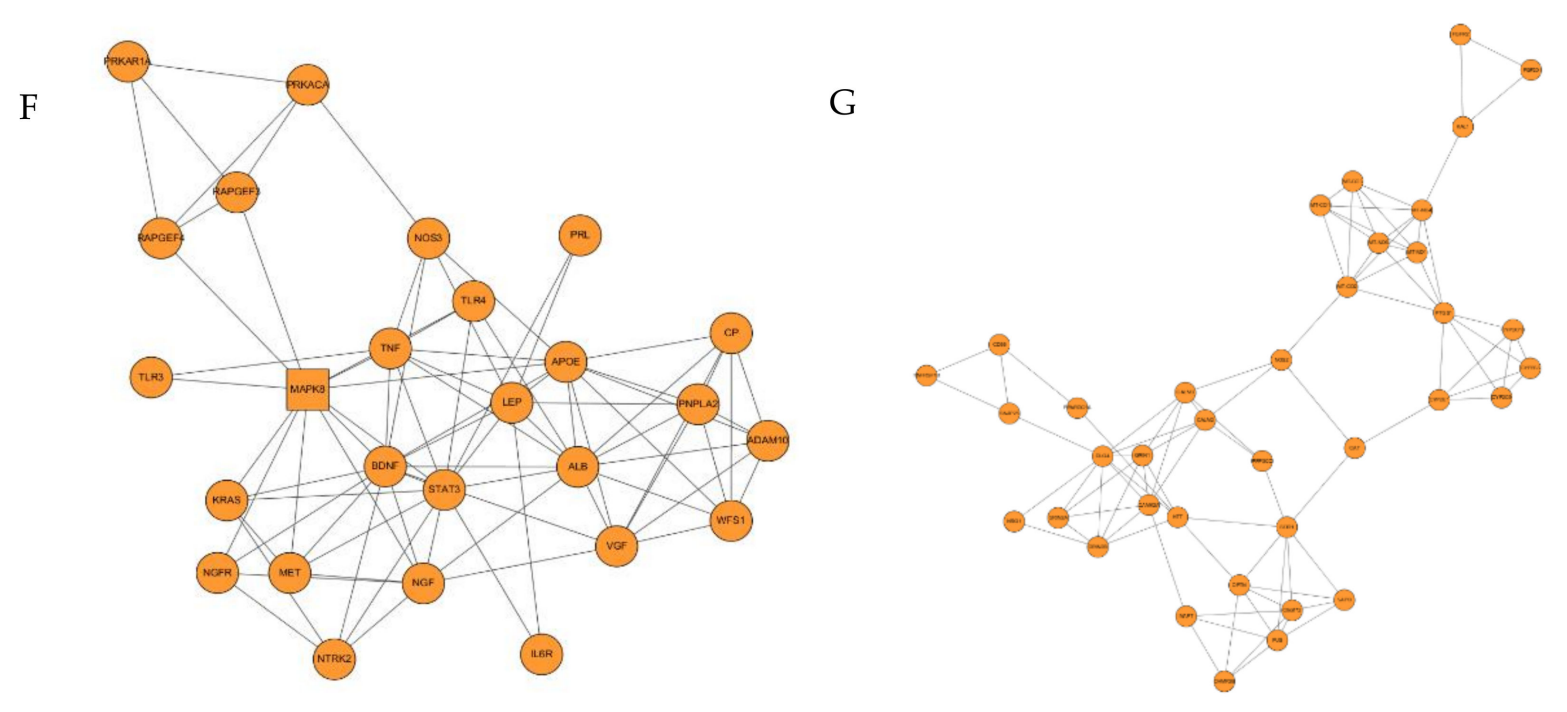
Clusters of DD target PPI network. (**A**–**G**) are clusters we found in the DD target PPI network which stands for cluster 1 to 7, respectively. The seed node of each clusters is presented as a square.

Seven clusters were found after DD target network was analyzed through MCODE (K-core = 5). This demonstrates that these clusters may be the most relevant to DD in studies at present. The details are described in Table 2 and Figure 4.

Cluster 1 contains 63 nodes and 1042 edges with a score of 33.613. The seed node of this cluster is PDYN (Proenkephalin-B) which compete with and mimic the effects of opiate drugs. It plays a role in a number of physiologic functions, including pain perception and responses to stress. PDYN is also involved in the regulation of chemical synaptic transmission and neuropeptide signaling pathway.

Cluster 2 contains 25 nodes and 200 edges with a score of 16.667. The seed node of this cluster is HTR4 (5-hydroxytryptamine receptor 4) which is one of the several different receptors for serotonin, a biogenic hormone that functions as a neurotransmitter, a hormone, and a mitogen. The activity of this receptor is mediated by G proteins that stimulate adenylate cyclase. It is involved in the regulation of chemical synaptic transmission, G protein-coupled receptor signaling pathway, and the regulation of appetite [16].

Cluster 3 contains 35 nodes and 187 edges with a score of 11. The seed node of this cluster is IL6 (Interleukin 6) which is a potent inducer of the acute phase response. It plays an essential role in the final differentiation of B-cells into Ig-secreting cells and induces nerve cell differentiation. IL6 is also involved in the regulation of neuroinflammatory response and negative regulation of neurogenesis. With meta-analysis results and many individual studies approved, depression is associated with the activation of the immune system, including increased expression of proinflammatory cytokines such as TNF-α and IL-6 [17]. 

Cluster 4 contains 38 nodes and 147 edges with a score of 7.946. The seed node of this cluster is NR1D1 (Nuclear receptor subfamily 1 group D member 1) which is related to metabolic, inflammatory and cardiovascular processes. It can also increase the hepatic expression of CYP7A1 via repression of NR0B2 and NFIL3 which are negative regulators of CYP7A1. 

Cluster 5 contains 11 nodes and 37 edges with a score of 7.4. The seed node of this cluster is PMS1 (PMS1 protein homolog 1). This protein is thought to be involved in the repair of DNA mismatches and the gene ontology (GO) annotations related to this gene include ATPase activity and mismatched DNA binding [18].

Cluster 6 contains 26 nodes and 84 edges with a score of 6.72. The seed node of this cluster is MAPK8 (Mitogen-activated protein kinase 8). Diseases associated with MAPK8 include fatty liver disease and renal fibrosis and the GO annotations related to this gene include transferase activity, transferring phosphorus-containing groups, and protein tyrosine kinase activity. Cluster 7 contains 37 nodes and 89 edges with a score of 4.944. There is no seed node presented in this cluster.

### 2.5. Analysis of HR Target-DD Target Network

#### 2.5.1. PPI Network of HR Targets and DD Targets

**Figure 5:**
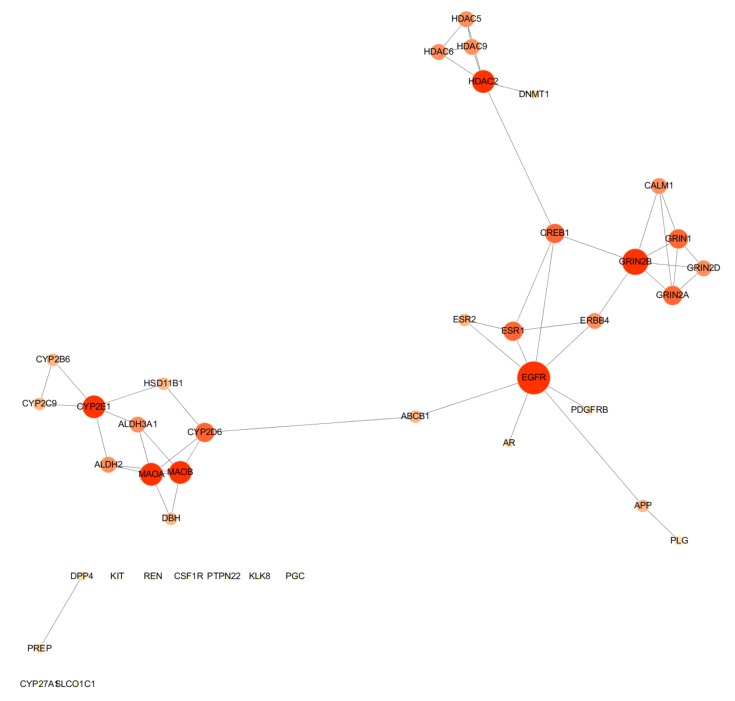
HR-DD PPI network. The closer, redder, and the larger the nodes are, the higher the degree of freedom they have.

After the intersection process, we found there is an overlap between HR and DD targets. As shown in Figure 5, the direct PPI was composed of 40 nodes and 49 edges. Specifically, 40 target genes were found and employed to create the HR-DD PPI network using Cytoscape and String with the confidence score cutoff set at 0.7. The top10 protein genes with the highest degree are EGFR, GRIN2B, HDAC2, MAOB, MAOA, CYP2E1, GRIN2A, CYP2D6, ESR1, and GRIN1, respectively.

#### 2.5.2. Clustering Analysis of HR-DD PPI Network

**Figure 6:**
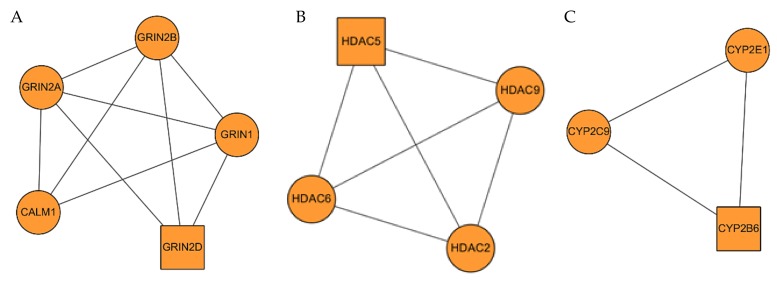
HR-DD PPI network. (**A**–**C**) are clusters we found in the HR-DD PPI network which stands for cluster 1 to 3, respectively. The seed node of each cluster is presented as a square.

Three clusters were obtained after conducting clustering analysis for HR-DD PPI network (K-core = 2). The details are described in Figure 6 and Table 3. Cluster 1 contains 5 nodes and 9 edges with a score of 4.5. The seed node of this cluster is GRIN2D (Glutamate ionotropic receptor NMDA type subunit 2D) which has been shown to be involved in long-term potentiation, an activity-dependent increase in the efficiency of synaptic transmission thought to underlie certain kinds of memory and learning. It is also involved in the regulation of synaptic plasticity [19] and modulation of chemical synaptic transmission. Cluster 2 contains 4 nodes and 6 edges with a score of 4. The seed node of this cluster is HDAC5 (Histone deacetylase 5) which is responsible for the deacetylation of lysine residues on the N-terminal part of the core histones (H2A, H2B, H3, and H4) and it is also involved in the regulation of inflammatory response. Cluster 3 contains 3 nodes and 3 edges with a score of 3. The seed node of this cluster is CYP2B6 (Cytochrome P450 2B6) which catalyze many reactions involved in drug metabolism and synthesis of cholesterol, steroids, and other lipids. It indicates that these aforementioned genes may be key to the treatment of depressive disorder with HR.

### 2.6. Potential Synergistic Mechanisms Analysis of HR Target-DD Target Network

#### 2.6.1. GO Enrichment Analysis

The major targets could be categorized into various functional modules by Gene Ontology enrichment analysis. GO enrichment analysis based on the topGO package [20]—available from the Bioconductor repository—was performed to identify the biological significance of the primary target with *p*-value cutoff set at 0.05 and the *p*-value were adjusted using the method introduced by Benjamini and Hochberg [21]. It controls the false discovery rate, the expected proportion of false discoveries among the rejected hypotheses. The false discovery rate is a less rigorous condition than the family wise error rate like Bonferroni correction [22,23,24], so this method is more powerful than the others.

**Figure 7:**
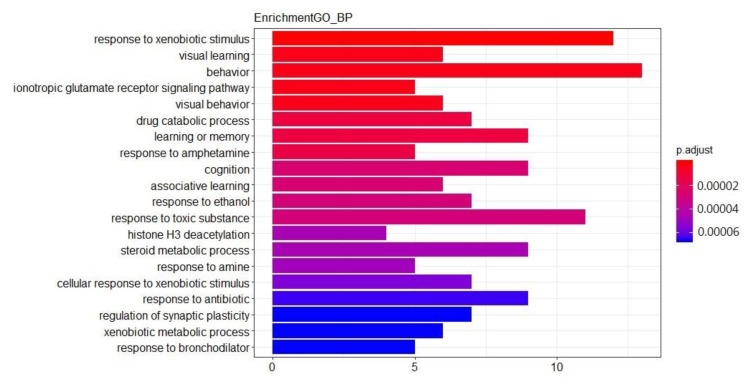
GO Enrichment—BP. Top 20 biology process from GO enrichment.

**Figure 8:**
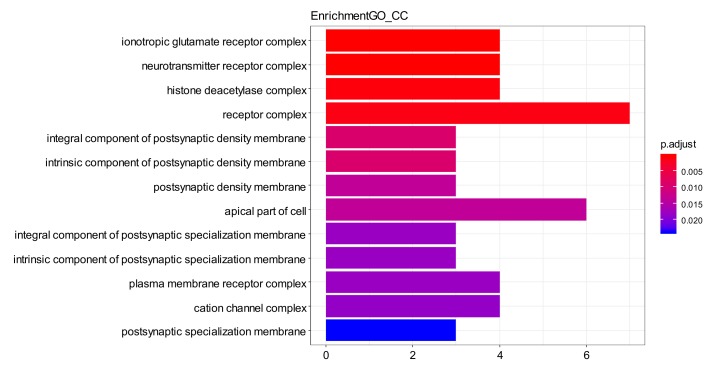
GO enrichment—CC. Top20 cellular component from GO enrichment.

**Figure 9:**
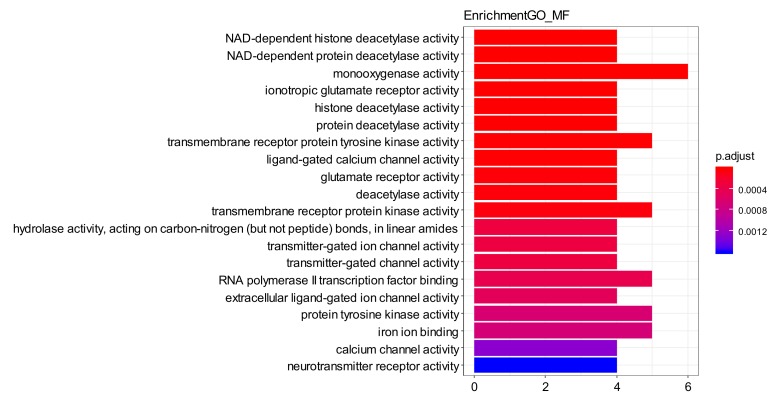
GO enrichment—MF. Top 20 molecular function from GO enrichment.

The GO enrichment analysis of the aforementioned network showed that total of 390 GO entries were obtained and top20 of each category were selected. Biology process (BP) is shown in Figure 7, including behavior (GO ID: 0007610), ionotropic glutamate receptor signaling pathway (GO ID: 0035235), regulation of synaptic plasticity (GO ID: 0048167), and response to amine (GO ID: 0014075). Cellular component (CC) is shown in Figure 8, including neurotransmitter receptor complex (GO ID: 0098878), ionotropic glutamate receptor complex (GO ID: 0008328), apical part of cell (GO ID: 0045177), intrinsic component of postsynaptic density membrane (GO ID: 0099146), and integral component of postsynaptic density membrane (GO ID: 0099061). Molecular function (MF) as shown in Figure 9, include monooxygenase activity (GO ID: 0004497), transmembrane receptor protein tyrosine kinase activity (GO ID: 0004714), and ionotropic glutamate receptor activity (GO ID: 0004970). These were all GO entries that play important parts in the central nervous system and affect mental diseases. The details of the above GO entries are described in Supplementary Table S1.

#### 2.6.2. Pathway Analysis to Explore the Therapeutic Mechanisms of HR on DD

**Figure 10:**
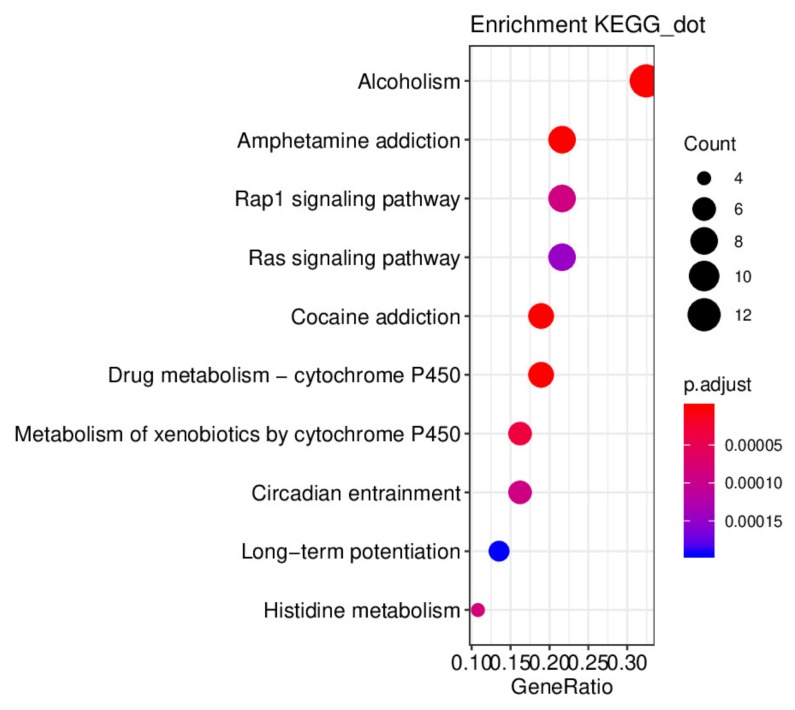
Pathway analysis. Dot plot of the top10 KEGG pathway.

To further verify that the biological process related to the target protein is associated with the occurrence of DD, a total of 40 signal pathways (*p*-value cutoff = 0.05) were screened and the top10 pathways were selected using Kyoto Encyclopedia of Genes and Genomes (KEGG) pathway analysis (Figure 10). Various pathways such as alcoholism pathway, amphetamine addiction pathway, cocaine addiction pathway, drug metabolism—cytochrome P450 pathway, metabolism of xenobiotics by cytochrome P450 pathway, long-term potentiation pathway, and histidine metabolism pathway were associated with those aforementioned targets. 

**Figure 11:**
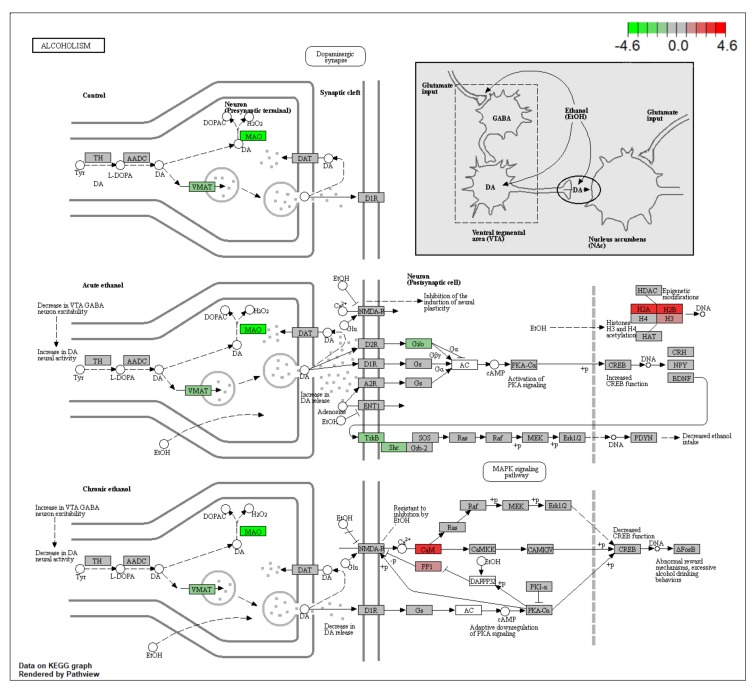
Pathway mapping of the alcoholism pathway. Green indicates down-regulation and red indicates up-regulation.

Based on the results of pathway analysis, it was found that these high-degree pathways are closely related to the central nervous system, major depressive disorder, Alzheimer’s disease, Parkinson’s disease, schizophrenia, and bipolar disorder. Especially, alcoholism (hsa05034) pathway exhibits the highest number of target connections (degree = 12), which includes consequential target genes such as MAOA, GRIN2A, GRIN2B, and CALM1. The results show that HR can down-regulate MAOA, MAOB, and simultaneously up-regulate CALM1 (Figure 11). MAOA and MAOB are crucial target genes of various mental diseases, and MAOA is particularly important in depression and anxiety [25,26]. MAOA is a major degrading enzyme—is encoded by the MAOA gene in humans—in the metabolic pathways of monoamine neurotransmitters such as norepinephrine, dopamine, and serotonin. Most of the antidepressants currently used involve the control of monoamine neurotransmitter turnover or monoamine receptor function. The initial action of mechanisms of most drugs used in the treatment of depression is to enhance central nervous system monoamine levels, particularly serotonin. Therefore, HR can ameliorate depression symptoms by down-regulating MAOA subsequently elevating serotonin and norepinephrine in the brain. 

Additionally, calcium (Ca^2+^) is one of the most significant intracellular messengers; the appropriate concentration of Ca^2+^ is necessary for neuronal excitability. When the Ca^2+^ concentration increases, Ca^2+^, calmodulin (CaM), and CaM kinase IIα (CaM KIIα) combine together to form the Ca^2+^-CaM-CaM KIIα signaling pathway, which is important in the plasticity of the central nervous system, learning and memory, mind, behavior, and other types of cognitive activities. In the previous 

Meta-analysis of genes associated with major depressive disorder, it showed that Calmodulin 1 (CALM1) plays an important role in the regulation in neurotransmission and calmodulin-related gene expression is altered in lateral habenula and frontal cortex of Major depressive disorder patients [27]. Therefore, HR can possibly ameliorate depression symptoms by increasing the expression of CALM1 and stimulating neurotransmission in the brain.

**Figure 12:**
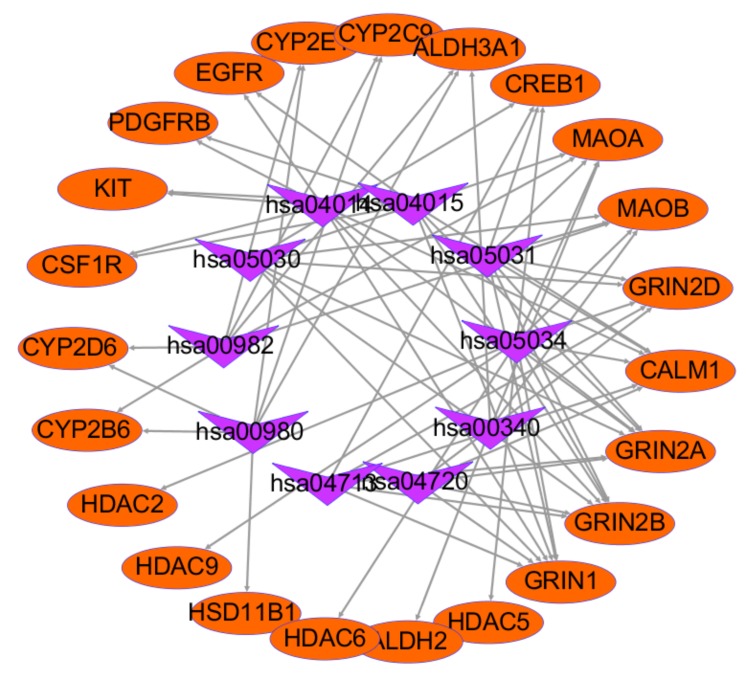
Target-pathway network of HR and DD. The purple arrow nodes are top10 KEGG pathway associated with *Hemerocallis* Radix and depression disorder targets, and the orange ellipse are the related targets.

The details of other related pathways are described in Table 4 and the target-pathway (T-P) network is shown in Figure 12, which contains 33 nodes including top10 KEGG pathways associated with 23 targets and 71 edges. The results show that glutamate ionotropic receptor NMDA type subunit family (GRIN1, GRIN2A, GRIN2B, and GRIN2D), monoamine oxidase family (MAOA and MAOB), and CALM1 accounted for the largest proportion. It indicates that these aforementioned genes may be key to the treatment of depressive disorder with HR.

## 3. Discussion

TCM might simultaneously target multiple physiological processes to arouse the human body’s potentiality to recover from unhealthiness. Unfortunately, the potential targets of Chinese herbs are difficult to be identified. The research strategy of network pharmacology provides a unique and innovative path for the study of TCM, including the mechanism of action of multi-component and multi-target. In the present study, we employed network pharmacology approaches to find potential bioactive ingredients from the herb HR associated with the important DD-related targets. By combining the advantages of data mining, machine learning, and neural network together, we predicted that multiple active components would target several proteins related to depressive disorder. 

**Figure 13:**
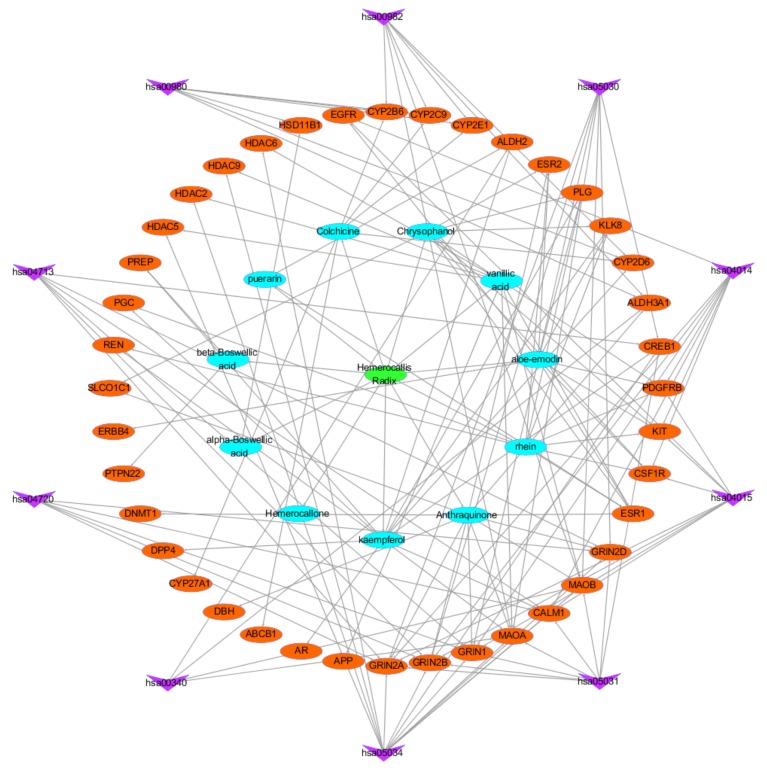
Component-target-pathway network of HR and DD. The green ellipse is the target herb— *Hemerocallis* Radix, the blue ellipse are bioactive components, the orange ellipse indicates the related targets, and the purple arrow nodes represent pathways.

Through the aforementioned analysis—pharmacokinetic analysis, C-T network, PPI network, GO enrichment and pathway analysis, T-P network, and cluster analysis—we found that the herb HR contained important candidate bioactive components for depression disorder treatment. To more intuitively determine the relation among component, target, and pathway, Figure 13 shows the results of the analysis of disease-component-target-pathway interactions.

*Hemerocallis* Radix has a beneficial effect in calming the spirit and even the temper, in order to reduce the feeling of melancholy. Pharmacological analysis has indicated that the active components in HR —aloe-emodin, α-boswellic acid, anthraquinone, β-Boswellic acid, chrysophanol, colchicine, hemerocallone, kaempferol, puerarin, rhein, and vanillic acid—impart beneficial effects on DD and related complications through the action of target proteins in various metabolic pathways. 

In this study, we try to interpret the synergistic effect of HR on DD from four aspects. First, C-T network showed that the average number of targets per component is 24.3, and the mean degree of components per target is 1.9. This clearly established that HR fits one of the most important characteristics of TCM—multi-component and multi-target. Second, PPI network recognized *EGFR, GRIN2B, HDAC2, MAOB, MAOA, CYP2E1, GRIN2A, CYP2D6, ESR1,* and *GRIN1* as hub genes and cluster analysis showed that *GRIN2D, HDAC5,* and *CYP2B6* are the center genes from three major clusters. PPI network unveiled the interaction between HR and DD-related targets, and found possibly significant targets in a more circumstantial point of view through the clustering method. Third, GO enrichment analysis indicated that all the targets interacting with the bioactive components of HR have a total of 390 GO entries and top20 of each category (BP, MF, CC) were selected. Most of these play important parts in the central nervous system that affect different steps of the synthesis or transportation of neurotransmitters. Finally, the pathway analysis proved that bioactive components of HR exert a synergistic effect on the treatment of depressive disorder through numerous pathways such as alcoholism pathway, amphetamine addiction pathway, Rap1 signaling pathway, Ras signaling pathway, drug metabolism—cytochrome P450 pathway, metabolism of xenobiotics by cytochrome P450 pathway, and so on. These pathways mostly down-regulate MAOA, MAOB, EGFR, and CYP2E1, while simultaneously up-regulating CALM1. This matches with the hub genes from PPI network, in which *EGFR* has been proved to be a significant depression-related gene [28], *MAOA* and *MAOB* are crucial target genes of various mental diseases, and MAOA is particularly important in depression and anxiety [25,26]. Moreover, CYP2E1, a member of the cytochrome P450 enzymes family, is referred to as toxicant metabolic enzyme in the liver cell [29]. Although it is mainly concentrated in liver cells, study proved that it has region, cell, and organelle specificity. CYP2E1—highly expressed in the brain microsomes—is an important member of catalyzing ethanol oxidation and is involved in the regulation of dopamine [30].

**Figure 14:**
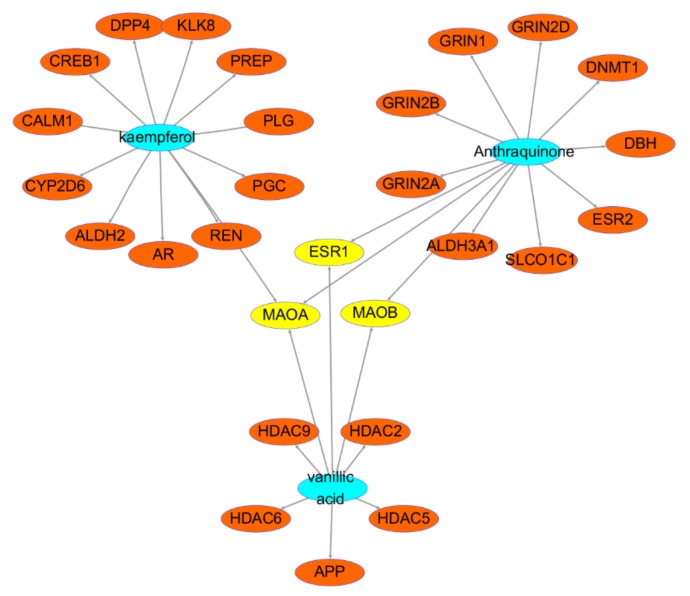
Top three key components of HR on DD treatment. The blue ellipse are the key bioactive components, the orange ellipse indicate the related targets, and the yellow ellipse represent the hub genes of the top3 key components.

Furthermore, the top3 key components and their related genes are presented in Figure 14. The main bioactive components are anthraquinone, kaempferol, and vanillic acid. Anthraquinone is an aromatic organic compound with formula C_14_H_8_O_2_. Previous studies showed that anthraquinone has only moderate inhibition effect or is even inactive against mouse Monoamine Oxidase (MAO) enzyme [31,32]. However, a recent study indicated that anthraquinone showed moderate to potent inhibition of human MAO enzyme activity. Though human MAO and mouse MAO have 92% sequence identity, differential sensitivity to phentermine inhibition suggests that structural and functional differences exist between them. That is to say, the type of MAO enzyme must be the reason for these inconsistent findings [33]. Consistent with our study, anthraquinone can down-regulate MAOA and MAOB, and all the gene data we used were set at Homo sapiens. Kaempferol is a natural flavonol—a type of flavonoid—found in a variety of plants and plant-derived foods. It has been proved to be a potent MAOA and MAOB inhibitor, antioxidant, and presents neuroprotective effect in the mouse model [34,35]. In spite of that, a more recent study showed that kaempferol acts as a selective inhibitor of human MAOA which is consistent with our study [36]. Vanillic acid is a phenolic acid with formula C_8_H_8_O_4_, found in some forms of vanilla and many other plant extracts. It is reported to possess strong antioxidant, anti-inflammatory, antinociceptive, and neuroprotective effects [37,38,39,40]. It has also been proved that vanillic acid has hepatoprotective effect, and is often used in the treatment of depression in TCM, and can suppress hepatic fibrosis in chronic liver injury [41]. Interestingly, although multiple phenolic acids have anti-depression effect, there is no scientific data that prove that vanillic acid alleviates depression, only several findings on the reduced concentrations of homo-vanillic acid (HVA) in cerebrospinal fluid [42,43,44] and mild inhibition of human MAOA and MAOB from virgin olive oil-extracted vanillic acid [45].

## 4. Materials and Methods 

### 4.1. Chemical Database Collection and Construction 

All components of HR were obtained from Traditional Chinese Medicine Systems Pharmacology Database and Analysis Platform (TCMSP, http://lsp.nwu.edu.cn/tcmsp.php) [46] and TCM-Mesh (http://mesh.tcm.microbioinformatics.org) [47]. TCMSP database contains 499 herbs, however, because of time limitations, there were components that had not been updated in the database. That is why TCM-Mesh, which contains 6235 herbs, was used as a verification and expansion tool for the component database construction. Finally, 26 components were collected from TCMSP and 2 components, Colchicine and Ethyl benzoate, were collected from TCM-Mesh. Further, the properties of components were retrieved from TCMSP, including molecular weight (MW), OB, Caco-2, DL, an octanol-water partition coefficient log P (AlogP), hydrogen bond donors (Hdon), hydrogen bond acceptors (Hacc), topological polar surface area (TPSA), rotatable bond number (RBN), and GI absorption was retrieved from SwissADME (http://www.swissadme.ch/index.php) [48].

### 4.2. Active Components Screening

ADME—absorption, distribution, metabolism, and excretion—is used in drug discovery to optimize the balance of properties necessary to convert leads into good medicines. The proper ADME screening can assure those candidates have better pharmacokinetic properties and hopefully, minimize drug–drug interactions in the future.

In this study, four ADME-related models were used to screen out the active components in HR, including OB, Caco-2, DL, and GI absorption. Beyond that, all screen-out components should follow Lipinski’s rule of five.

Lipinski’s rule of five is a rule of thumb to evaluate if a chemical compound with certain pharmacological or biological activities could be a likely orally active drug in humans. In this rule, compounds who met the requirements of MW, Hdon, Hacc, AlogP, and RBN seemed to be more possible to become a drug [49,50]. 

OB is defined as “the rate and extent to which the active ingredient or active moiety is absorbed from a drug product and becomes available at the site of action” by the Food and Drug Administration (FDA) [51]. It portrays the percentage of an orally administered drug that absorbs and reaches human body circulation. High oral bioavailability was often a key indicator to determine the drug-like property of bioactive molecules as therapeutic agents [52]. In the United States, FDA indicates the importance of bioavailability and this information became part of the drug development and regulatory processes in a New Drug Application [53]. In this study, those components with suitable OB ≥ 30% were selected as candidate components for further research.

Human intestinal cell line Caco-2 is a most common tool to study the passive diffusion of drugs across the intestinal epithelium. The Caco-2 cell model is a human-cloned colonic adenocarcinoma cell that is structurally and functionally similar to intestinal epithelial cells. The transport rates of components (nm/s) in Caco-2 monolayers represents the intestinal epithelial permeability [46]. Those components with Caco-2 > −0.4 were selected as candidate components, because components with Caco-2 < −0.4 are not permeable.

DL is an established concept for drug design that is used to estimate which compounds have the “drug-like” properties. The DL values of these components were calculated by the database-dependent DL evaluation approach based on Tanimoto coefficient, which equation is showing below. In this equation, A represents the molecular descriptor of herbal components, and B is the average molecular property of all components in DrugBank (http://www.drugbank.ca/) [54]. The threshold of DL was set to 0.18, which is used as a selection criterion for “druglike” compounds in the traditional Chinese herbs [55]. Any component that does not meet this criterion will be screen out from this database.

(1) $$T\;\left(A,\;B\right)\;=\frac{\left(A\;\times \;B\right)}{\left({\left|A\right|}^{2}\;+\;{\left|B\right|}^{2}\;-\;A\;\times \;B\right)}$$

Gastrointestinal (GI) absorption is a pharmacokinetic behavior crucial to estimate the various stages of the drug discovery processes, which can be calculated by an accurate predictive model, the Brain Or IntestinaL EstimateD permeation method (BOILED-Egg) [56]. This model uses membrane permeation-related characteristics, lipid solubility—WlogP (n-octanol/water partition coefficient; Y-axis) and polarity—TPSA (topological polar surface area; X-axis) to calculate its GI absorption value, and can be found on the website SwissADME [48]. The screening criterion of GI absorption was defined as high. Based on this analysis ten components were excluded from further analysis.

Lastly, there are some screen-out components we add back into the database. Vanillic acid, puerarin, α-Boswellic acid, β-Boswellic acid and Chrysophanol were added back into the list because numerous studies proved that they are beneficial for hepatoprotection [41,55,57,58]. Even though the DL of anthraquinone is lower than 0.18, it has become a member of this database again nevertheless for the reason that it is one of the main active ingredients in HR [59]. Taken together, 28 components obtained from TCMSP and TCM-Mesh remained 11.

### 4.3. Targets fishing

#### 4.3.1. Identified and Predicted Targets of Hemerocallis Radix

To obtain the target of active components in HR, the commonly used databases, i.e., HitPick [60], similarity ensemble approach (SEA) [61], and PubChem [62] were engaged to collect the targets. All chemical structures were prepared and converted into canonical SMILES using Open Babel Toolkit (version 2.4.1). Known therapeutic targets of HR were collected from PubChem, while HitPick and SEA were applied for the use of target prediction.

#### 4.3.2. Targets of Depressive Disorder

The DD targets were gathered from the DisGeNET database (http://www.disgenet.org/) [63], which offers information about disease targets. The keywords “depressive disorder” were used, and the targets were human genes/proteins enrolled in this study (DisGeNET UMLS CUI: C0011581). A total of 740 DD targets were gathered, and furthermore, using the String plugin [64] in Cytoscape 3.7.1 a PPI network was built in which the confidence score cutoff was set at 0.7. Hence, the numbers of 709 DD targets were obtained.

### 4.4. Network Construction and Clustering Analysis

Artificial neural network was constructed through the network visualization software Cytoscape, which is used to visualize and analyze the molecular interactions and biological pathway. Draw Venn Diagram (http://bioinformatics.psb.ugent.be/webtools/Venn/) was applied to calculate the intersection between HR and DD targets. 

To study further into the network, MCODE plugin was introduced to generate clusters [65]. This tool can identify the major hubs of HR against DD by setting its K-core, which filters out clusters that do not contain a maximally inter-connected sub-cluster of at least k degrees. Increasing the value of K-core will generate fewer clusters and exclude smaller clusters. Nevertheless, we can find the highest scoring node, called seed which is represented as a square. The seed might have a chance of becoming the key target of this cluster.

### 4.5. Gene Ontology and Pathway Enrichment Analysis

The software R language with a graphical user interface, RGui (Version 3.6.1) was adopted to analyze the representative biological processes and pathways associated with HR against DD. All obtained targets from previous preparation were imported. Total of 390 GO entries were found and top20 entries of each category, namely BP, CC, and MF, were obtained with the *p*-value set at 0.05. 

The latest pathway data were obtained from the KEGG database [66] for KEGG pathway enrichment analyses. *p*-values were set at 0.05 as the cut-off criterion. A total of 40 pathways were screened and top10 pathways were selected. The results of the analysis were annotated by Pathview [67] in the R Bioconductor package (https://www.bioconductor.org/).

### 4.6. Component-Target-Pathway Network Construction

In this research, the network model of "component-target-pathway" interaction was established through Cytoscape3.7.1. In network interactions, nodes represent components, targets, and pathways, whereas edges represent the interaction of each other. Based on this neural network model, the pathway of active components and targets in depressive disorder is initially explored to provide a preliminary theoretical basis for the design of subsequently targeted drugs. 

## 5. Conclusions

The aforementioned results highlight that HR has intervention effects on DD through multiple bioactive compounds and targets. In conclusion, anthraquinone, kaempferol, and vanillic acid as the main bioactive components can alleviate depression symptoms by down-regulated MAOs. While there is no literature that shows the effect of anthraquinone and vanillic acid on ESR1, studies showed that it acts as an important factor of depression especially in women [68,69,70]. ESR1 can regulate neurotransmitters including increasing the concentration of serotonin and norepinephrine, and it is involved in the regulation of the number and function of serotonin receptors, thereby controlling the activity of serotonin-activated neurons [71,72]. 

In the present study, a network-based computational strategy was established to uncover the pharmacological mechanism of the compounds of *Hemerocallis* Radix on depressive disorder. It will provide new ideas for further research on ethnopharmacology, Traditional Chinese Medicinal herbs, and ethnic compounds. The targets, clusters, biological processes, and pathways associated with HR were discovered through this study. HR target-DD target network exhibited the effective bioactive components, potential pharmacology, and molecular mechanism of HR for treating DD. This study provides a suitable combination strategy to uncover the therapeutic mechanism of multi-component drugs on a systematic level, which lays the foundation for the clinical application.

The alcoholism pathway is regulated at different levels by various mechanism and can crosstalk with other signaling pathways. The results of our study support the role of anthraquinone, kaempferol, and vanillic acid on MAOA, MAOB, and ESR1 in the etiology of depressive disorder. Many studies focusing medicinal herbs were carried out with animal models. However, several researches suggested that it shows different results in humans, indicating the importance of assessing the effect of the abovementioned active components on specific target toward the treatment of depressive disorder in human. While there is no ample evidence of the effect of anthraquinone and vanillic acid on ESR1, further research in this area may reveal details in assigning causative relationships.

## Figures and Tables

**Table 1:** The information of active components in *Hemerocallis* Radix (HR).

ID	Molecule Name	MW	AlogP	nHdon	nHacc	TPSA	OB	Caco-2	DL	GI Absorption	Lipinski’s Rule
HR01	Aloe-emodin	270.25	1.67	3	5	94.83	83.38	−0.12	0.24	High	Yes
HR02	α-Boswellic acid	456.78	6.42	2	3	57.53	39.32	0.6	0.75	Low	Yes
HR03	Anthraquinone	208.22	2.81	0	2	34.14	56.1	0.86	0.14	High	Yes
HR04	β-Boswellic acid	456.78	6.47	2	3	57.53	39.55	0.59	0.75	Low	Yes
HR05	Chrysophanol	254.25	2.76	2	4	74.6	18.64	0.62	0.21	High	Yes
HR06	Colchicine	385.45	1.47	2	7	94.09	39.34	0.12	0.57	High	Yes
HR07	Hemerocallone	356.35	2.59	0	7	76.36	63.01	0.77	0.54	High	Yes
HR08	Kaempferol	286.25	1.77	4	6	111.13	41.88	0.26	0.24	High	Yes
HR09	Puerarin	416.41	−0.06	6	9	160.82	24.03	−1.15	0.69	High	Yes
HR10	Rhein	284.23	1.88	3	6	111.9	47.07	−0.2	0.28	High	Yes
HR11	Vanillic acid	168.16	1.15	2	4	66.76	35.47	0.43	0.04	High	Yes

**Table 2:** Clusters of DD target PPI network.

Cluster	Score	Nodes	Edges	Gene IDs
1	33.613	63	1042	ADRA2C, CNR2, ADRA2A, GNRH1, CNR1, GNA11, AGTR1, PIK3CA, CHRM2, NTS, CX3CR1, ADRBK1, OPRK1, TAC3, GNRHR, TAC1, POMC, KISS1, GALR3, GALR2, EDN1, HCRT, AVPR1B, HCRTR1, PYY, GAL, AVP, GNAQ, OXTR, PNOC, NPS, NPY, CXCL8, KISS1R, DRD2, DRD3, OXT, DRD4, GRM1, GRM3, GRM5, GRM7, HTR1D, HTR1A, HTR1B, TACR3, F2RL3, TACR1, OPRM1, GRPR, HTR2C, NPSR1, HTR2A, TRH, ADCY1, ADCY8, NPY1R, ADCY7, PROK2, PENK, ADCY5, PROKR2, PDYN
2	16.667	25	200	NMS, DRD1, ADRB1, AGT, ADRB2, CRH, DRD5, MCHR1, MC4R, PMCH, HTR4, ADCYAP1R1, ADCYAP1, HTR6, HTR7, CRHR1, CASR, CRHR2, GNB1, APP, GNAS, GNB3, TAAR6, VIPR2, MC1R
3	11	35	187	MAPK1, SERPING1, MAPK3, AP2B1, ORM1, CSF3, CSF2, M6PR, FGF2, PDGFB, CLU, PLG, IL17A, SIRT1, TP53, TGFB1, UBQLN2, IL10, ESR1, CREB1, IL13, IFNG, IL18, ARRB1, ARRB2, IL4, IL1A, IL6, PTGS2, IL1B, OCRL, A2M, HGS, IGF1, INS
4	7.946	38	147	AR, STIP1, ERBB4, CALM1, CLOCK, VEGFA, PER2, PER1, CRP, PER3, RORA, NOS1, MAPK14, GATA3, RAC1, ATF2, NR3C1, SERPINE1, HSP90AB1, NTRK1, NR3C2, ADIPOQ, CRY2, CRY1, PDGFRB, FKBP4, KIT, FKBP5, AKT1, NR1D1, EGFR, HSP90AA1, NTF3, TIMELESS, ARNTL, FGF13, NPAS2, FGFR1
5	7.4	11	37	CDKN2A, BRCA1, OGG1, ATM, MSH6, RFC2, PMS2, MSH2, MLH1, MLH3, PMS1
6	6.72	26	84	PRKAR1A, STAT3, MET, BDNF, NOS3, PRL, WFS1, TNF, PNPLA2, ALB, KRAS, VGF, NTRK2, RAPGEF3, RAPGEF4, NGFR, IL6R, ADAM10, LEP, PRKACA, CP, TLR4, APOE, TLR3, MAPK8, NGF
7	4.944	37	89	TNFRSF1B, KAL1, CALM3, CYP2E1, CALM2, OPTN, NOS2, GRIN1, C9orf72, FUS, FGF20, PPP3CC, GRIN2A, SOD1, CYP2B6, CD36, MT-CO3, SNAP25, MT-CO2, MT-CO1, PPARGC1A, CAT, MT-ND1, HTT, VAPB, MT-ND4, MAPT, DLG4, GRIN2B, PTGS1, CYP2C9, MT-ND6, CHMP2B, CAMK2A, NRG1, FGFR2, CYP2C19

**Table 3:** Clusters of HR-DD PPI network.

Cluster	Score	Nodes	Edges	Gene IDs
1	4.5	5	9	GRIN2D, GRIN1, GRIN2B, CALM1, GRIN2A
2	4	4	6	HDAC5, HDAC6, HDAC9, HDAC2
3	3	3	3	CYP2B6, CYP2E1, CYP2C9

**Table 4:** Pathways associated with 40 candidate targets according to enrichment analysis based on KEGG.

ID	Pathway	*p*-Value	p.adjust	Count	Gene IDs
hsa05034	Alcoholism	1.95 × 10^−11^	2.84 × 10^−9^	12	MAOB/MAOA/HDAC5/CREB1/GRIN2D/GRIN1/HDAC2/HDAC9/GRIN2B/GRIN2A/CALM1/HDAC6
hsa05031	Amphetamine addiction	6.17 × 10^−10^	4.51 × 10^−8^	8	MAOB/MAOA/CREB1/GRIN2D/GRIN1/GRIN2/GRIN2A/CALM1
hsa04015	Rap1 signaling pathway	4.29 × 10^−6^	8.94 × 10^−5^	8	EGFR/GRIN1/PDGFRB/KIT/GRIN2B/GRIN2A/CSF1R/CALM1
hsa04014	Ras signaling pathway	8.96 × 10^−6^	1.45 × 10^−4^	8	EGFR/GRIN1/PDGFRB/KIT/GRIN2B/GRIN2A/CSF1R/CALM1
hsa05030	Cocaine addiction	2.00 × 10^−9^	9.73 × 10^−8^	7	MAOB/MAOA/CREB1/GRIN2D/GRIN1/GRIN2B/GRIN2A
hsa00982	Drug metabolism - cytochrome P450	3.18 × 10^−8^	1.16 × 10^−6^	7	MAOB/CYP2D6/MAOA/ALDH3A1/CYP2B6/CYP2C9/CYP2E1
hsa00980	Metabolism of xenobiotics by cytochrome P450	1.18 × 10^−6^	3.44 × 10^−5^	6	CYP2D6/ALDH3A1/CYP2B6/CYP2C9/CYP2E1/HSD11B1
hsa04713	Circadian entrainment	4.97 × 10^−6^	9.06 × 10^−5^	6	CREB1/GRIN2D/GRIN1/GRIN2B/GRIN2A/CALM1
hsa04720	Long-term potentiation	1.32 × 10^−5^	1.93 × 10^−4^	5	GRIN2D/GRIN1/GRIN2B/GRIN2A/CALM1
hsa00340	Histidine metabolism	3.36 × 10^−6^	8.18 × 10^−5^	4	MAOB/MAOA/ALDH2/ALDH3A1

## Supplementary Materials

Supplementary materials can be found at https://www.mdpi.com/1422-0067/21/5/1868/s1.

## Abbreviations

AGT	Angiotensinogen
AKT1	AKT Serine/Threonine Kinase 1
AlogP	An Octanol-Water Partition Coefficient log P
APP	Amyloid Beta Precursor Protein
BOILED-Egg	Brain or Intestinal Estimated Permeation Method
BP	Biology Process
Caco-2	Caco-2 Permeability
CALM1	Calmodulin 1
CC	Cellular Component
C-T	Component-Target
CYP2B6	Cytochrome P450 2B6
CYP2D6	Cytochrome P450 Family 2 Subfamily D Member 6
CYP2E1	Cytochrome P450 Family 2 Subfamily E Member 1
DD	Depressive Disorder
DL	Drug-Likeness
EGFR	Epidermal Growth Factor Receptor
ESR1	Estrogen Receptor 1
GNB1	G Protein Subunit Beta 1
GNB3	G Protein Subunit Beta 3
GO	Gene Ontology
GRIN1	Glutamate Ionotropic Receptor NMDA Type Subunit 1
GRIN2A	Glutamate Ionotropic Receptor NMDA Type Subunit 2A
GRIN2B	Glutamate Ionotropic Receptor NMDA Type Subunit 2B
GRIN2D	Glutamate Ionotropic Receptor NMDA Type Subunit 2D
Hacc	Hydrogen Bond Acceptors
HDAC2	Histone Deacetylase 2
HDAC5	Histone Deacetylase 5
Hdon	Hydrogen Bond Donors
HR	Hemerocallis Radix
HTR4	5-Hydroxytryptamine Receptor 4
HVA	Homovanillic Acid
IL6	Interleukin 6
INS	Insulin
KEGG	Kyoto Encyclopedia of Genes and Genomes
MAOA	Monoamine Oxidase A
MAOB	Monoamine Oxidase B
MAPK8	Mitogen-Activated Protein Kinase 8
MF	Molecular Function
MW	Molecular Weight
NR1D1	Nuclear Receptor Subfamily 1 Group D Member 1
OB	Oral Bioavailability
PDYN	Proenkephalin-B
PIK3CA	Phosphatidylinositol-4,5-Bisphosphate 3-Kinase Catalytic Subunit Alpha
PMS1	PMS1 Protein Homolog 1
PPI	Protein-Protein Interaction
RBN	Rotatable Bond Number
SEA	Similarity Ensemble Approach
TCM	Traditional Chinese Medicine
TCMSP	Traditional Chinese Medicine Systems Pharmacology Database and Analysis Platform
T-P	Target-Pathway
TP53	Tumor Protein P53
TPSA	Topological Polar Surface Area
