# Supplementary material for: Reveals of New Candidate Active Components in Hemerocallis Radix and Its Anti-Depression Action of Mechanism Based on Network Pharmacology Approach
Source: Int J Mol Sci. 2020 Mar 9;21(5):1868. doi: 10.3390/ijms21051868 (PMC7084327; doi:10.3390/ijms21051868)
Supplement: Supplementary file 1 [file ijms-21-01868-s001.pdf]

Table S1. Enrichment analysis of HR-DD targets based on Gene Ontology (GO) annotation.

Ontology	ID	Term	*p* value	p.adjust	Count	Gene IDs
BP	GO:0009410	Response to xenobiotic stimulus	1.83×10^{-11}	3.42×10^{-8}	12	CYP2D6/EGFR/HDAC5/ALDH3A1/GRIN1/HDAC2/DBH/HDAC9/CYP2B6/CYP2C9/CYP2E1/GRIN2A
BP	GO:0008542	Visual learning	4.80×10^{-9}	3.06×10^{-6}	6	CREB1/GRIN1/DBH/KIT/GRIN2A/APP
BP	GO:0007610	Behavior	6.12×10^{-9}	3.06×10^{-6}	13	EGFR/DPP4/KLK8/CREB1/GRIN2D/GRIN1/HDAC2/DBH/KIT/GRIN2B/REN/GRIN2A/APP
BP	GO:0035235	Ionotropic glutamate receptor signaling pathway	6.55×10^{-9}	3.06×10^{-6}	5	GRIN2D/GRIN1/GRIN2B/GRIN2A/APP
BP	GO:0007632	Visual behavior	1.01×10^{-8}	3.76×10^{-6}	6	CREB1/GRIN1/DBH/KIT/GRIN2A/APP
BP	GO:0042737	Drug catabolic process	3.92×10^{-8}	1.22×10^{-5}	7	MAOB/CYP2D6/MAOA/ALDH2/DBH/CYP2B6/CYP2C9
BP	GO:0007611	Learning or memory	4.71×10^{-8}	1.26×10^{-5}	9	EGFR/KLK8/CREB1/GRIN1/DBH/KIT/GRIN2B/GRIN2A/APP
BP	GO:0001975	Response to amphetamine	5.86×10^{-8}	1.37×10^{-5}	5	GRIN1/HDAC2/DBH/HDAC9/GRIN2A
BP	GO:0050890	Cognition	1.26×10^{-7}	2.60×10^{-5}	9	EGFR/KLK8/CREB1/GRIN1/DBH/KIT/GRIN2B/GRIN2A/APP
BP	GO:0008306	Associative learning	1.39×10^{-7}	2.60×10^{-5}	6	CREB1/GRIN1/DBH/KIT/GRIN2A/APP
BP	GO:0045471	Response to ethanol	1.64×10^{-7}	2.78×10^{-5}	7	MAOB/GRIN1/HDAC2/DBH/GRIN2B/CYP2E1/GRIN2A
BP	GO:0009636	Response to toxic substance	1.82×10^{-7}	2.83×10^{-5}	11	MAOB/CREB1/GRIN1/HDAC2/DBH/HDAC9/PDGFRB/GRIN2B/CYP2E1/GRIN2A/HDAC6
BP	GO:0070932	Histone H3 deacetylation	3.16×10^{-7}	4.54×10^{-5}	4	HDAC5/HDAC2/HDAC9/HDAC6
BP	GO:0008202	Steroid metabolic process	3.41×10^{-7}	4.55×10^{-5}	9	CYP2D6/CYP27A1/KIT/CYP2B6/CYP2C9/CYP2E1/ESR1/HSD11B1/APP
BP	GO:0014075	Response to amine	3.83×10^{-7}	4.77×10^{-5}	5	GRIN1/HDAC2/DBH/HDAC9/GRIN2A
BP	GO:0071466	Cellular response to xenobiotic stimulus	4.78×10^{-7}	5.58×10^{-5}	7	CYP2D6/EGFR/ALDH3A1/GRIN1/CYP2B6/CYP2C9/CYP2E1
BP	GO:0046677	Response to antibiotic	5.85×10^{-7}	6.44×10^{-5}	9	MAOB/GRIN1/HDAC2/DBH/PDGFRB/GRIN2B/CYP2E1/GRIN2A/HDAC6

BP	GO:0048167	Regulation of synaptic plasticity	6.68×10^{-7}	6.65×10^{-5}	7	CREB1/GRIN2D/GRIN1/KIT/GRIN2B/GRIN2A/APP
BP	GO:0006805	Xenobiotic metabolic process	6.84×10^{-7}	6.65×10^{-5}	6	CYP2D6/ALDH3A1/GRIN1/CYP2B6/CYP2C9/CYP2E1
BP	GO:0097366	Response to bronchodilator	7.47×10^{-7}	6.65×10^{-5}	5	GRIN1/HDAC2/DBH/HDAC9/GRIN2A
CC	GO:0008328	Ionotropic glutamate receptor complex	1.30×10^{-6}	0.000173	4	GRIN2D/GRIN1/GRIN2B/GRIN2A
CC	GO:0098878	Neurotransmitter receptor complex	1.82×10^{-6}	0.000173	4	GRIN2D/GRIN1/GRIN2B/GRIN2A
CC	GO:0000118	Histone deacetylase complex	9.67×10^{-6}	0.000612	4	HDAC5/HDAC2/HDAC9/HDAC6
CC	GO:0043235	Receptor complex	1.94×10^{-5}	0.000922	7	ERBB4/EGFR/GRIN2D/GRIN1/GRIN2B/GRIN2A/APP
CC	GO:0099061	Integral component of postsynaptic density membrane	0.00024	0.008987	3	ERBB4/GRIN1/GRIN2A
CC	GO:0099146	Intrinsic component of postsynaptic density membrane	0.000284	0.008987	3	ERBB4/GRIN1/GRIN2A
CC	GO:0098839	Postsynaptic density membrane	0.000508	0.013339	3	ERBB4/GRIN1/GRIN2A
CC	GO:0045177	Apical part of cell	0.000562	0.013339	6	EGFR/DPP4/ABCB1/PDGFRB/REN/APP
CC	GO:0099060	Integral component of postsynaptic specialization membrane	0.000869	0.017824	3	ERBB4/GRIN1/GRIN2A
CC	GO:0098948	Intrinsic component of postsynaptic specialization membrane	0.000967	0.017824	3	ERBB4/GRIN1/GRIN2A
CC	GO:0098802	Plasma membrane receptor complex	0.001032	0.017824	4	GRIN2D/GRIN1/GRIN2B/GRIN2A
CC	GO:0034703	Cation channel complex	0.001156	0.018303	4	GRIN2D/GRIN1/GRIN2B/GRIN2A
CC	GO:0099634	Postsynaptic specialization membrane	0.001627	0.023783	3	ERBB4/GRIN1/GRIN2A
MF	GO:0017136	NAD-dependent histone deacetylase activity	9.76×10^{-8}	1.05×10^{-5}	4	HDAC5/HDAC2/HDAC9/HDAC6
MF	GO:0034979	NAD-dependent protein deacetylase activity	1.36×10^{-7}	1.05×10^{-5}	4	HDAC5/HDAC2/HDAC9/HDAC6

MF	GO:0004497	Monooxygenase activity	1.70×10^{-7}	1.05×10^{-5}	6	CYP2D6/CYP27A1/DBH/CYP2B6/ CYP2C9/CYP2E1
MF	GO:0004970	Ionotropic glutamate receptor activity	1.85×10^{-7}	1.05×10^{-5}	4	GRIN2D/GRIN1/GRIN2B/GRIN2A
MF	GO:0004407	Histone deacetylase activity	3.22×10^{-7}	1.45×10^{-5}	4	HDAC5/HDAC2/HDAC9/HDAC6
MF	GO:0033558	Protein deacetylase activity	4.13×10^{-7}	1.55×10^{-5}	4	HDAC5/HDAC2/HDAC9/HDAC6
MF	GO:0004714	Transmembrane receptor protein tyrosine kinase activity	5.54×10^{-7}	1.79×10^{-5}	5	ERBB4/EGFR/PDGFRB/KIT/CSF1R
MF	GO:0099604	Ligand-gated calcium channel activity	6.50×10^{-7}	1.84×10^{-5}	4	GRIN2D/GRIN1/GRIN2B/GRIN2A
MF	GO:0008066	Glutamate receptor activity	1.18×10^{-6}	2.96×10^{-5}	4	GRIN2D/GRIN1/GRIN2B/GRIN2A
MF	GO:0019213	Deacetylase activity	1.41×10^{-6}	3.19×10^{-5}	4	HDAC5/HDAC2/HDAC9/HDAC6
MF	GO:0019199	Transmembrane receptor protein kinase activity	2.13×10^{-6}	4.37×10^{-5}	5	ERBB4/EGFR/PDGFRB/KIT/CSF1R
MF	GO:0016811	Hydrolase activity, acting on carbon-nitrogen (but not peptide) bonds, in linear amides	1.56×10^{-5}	0.000294	4	HDAC5/HDAC2/HDAC9/HDAC6
MF	GO:0022824	Transmitter-gated ion channel activity	1.87×10^{-5}	0.000302	4	GRIN2D/GRIN1/GRIN2B/GRIN2A
MF	GO:0022835	Transmitter-gated channel activity	1.87×10^{-5}	0.000302	4	GRIN2D/GRIN1/GRIN2B/GRIN2A
MF	GO:0001085	RNA polymerase II transcription factor binding	2.64×10^{-5}	0.000398	5	HDAC5/CREB1/HDAC2/ESR1/AR
MF	GO:0005230	Extracellular ligand-gated ion channel activity	3.34×10^{-5}	0.000472	4	GRIN2D/GRIN1/GRIN2B/GRIN2A
MF	GO:0004713	Protein tyrosine kinase activity	4.76×10^{-5}	0.000633	5	ERBB4/EGFR/PDGFRB/KIT/CSF1R
MF	GO:0005506	Iron ion binding	5.40×10^{-5}	0.000678	5	CYP2D6/CYP27A1/CYP2B6/CYP2C 9/CYP2E1
MF	GO:0005262	Calcium channel activity	0.000108	0.001286	4	GRIN2D/GRIN1/GRIN2B/GRIN2A
MF	GO:0030594	Neurotransmitter receptor activity	0.000142	0.001599	4	GRIN2D/GRIN1/GRIN2B/GRIN2A
